# Diet in Infantile Eczema
*On Eczema Infantile. By Erasmus Wilson, F.R.S. London: Richards. 1856.


**Published:** 1857-01

**Authors:** 


### DIET IN INFANTILE ECZEMA.*

Mr. Wilson recommends the following diet in this very troublesome affection:—"The diet of the child, while under this treatment, must be carefully inquired into; it should be good, wholesome, and nutritious. The leading constitutional indication is to nourish properly; and this idea should be carried out in the food as well as in the medicine. I find the juice of meat of great value in these cases, and it may be given either alone, as beef or mutton tea, or mixed with the other food. The consideration of diet and food brings me to an important dietetic medicine, which is of great value in this disease, when the latter is attended with emaciation, and in the chronic stage; in acute cases it is less applicable; I mean the cod-liver oil. The child will often take the oil greedily in its natural state, and its good effects on nutrition are speedily made apparent: it may be given with safety to the youngest infant. In children somewhat older, and particularly in chronic cases, the cod-liver oil chocolate becomes a useful ingredient of diet."

### TREATMENT OF PULMONARY CONSUMPTION.†

Although this book was written long ago, it is new to us; and we notice it specially, because it is the express wish of

* On Eczema Infantile. By Erasmus Wilson, F.R.S. London: Richards. 1856.

